# Anthelmintic resistance of horse strongyle nematodes to fenbendazole in Lithuania

**DOI:** 10.1186/s13028-022-00645-y

**Published:** 2022-09-15

**Authors:** Evelina Dauparaitė, Tomas Kupčinskas, Marian Varady, Saulius Petkevičius

**Affiliations:** 1 Department of Veterinary Pathobiology, Veterinary Academy, Lithuanian University of Health Sciences, Tilzes str. 18, 47181 Kaunas, Lithuania; 2 Institute of Parasitology, Slovak Academy of Sciences, Hlinkova 3, Kosice, Slovakia

**Keywords:** *Cyathostominae*, FECRT, In vivo, Mini-FLOTAC

## Abstract

**Background:**

Control of strongyle infections presents a global challenge for horse practitioners due to the development of anthelmintic resistance (AR), however comprehensive information on AR in Lithuania is still lacking. The aim of this study was to assess the current situation of fenbendazole (FBZ) AR in horses at stable level in Lithuania.

**Results:**

Faecal samples from 121 horses from six stables were examined using the Mini-FLOTAC method. Of these, 89 horses met the inclusion criteria that included strongyle faecal egg counts (FEC) exceeding 200 eggs per gram (EPG). Faecal egg count reduction tests (FECRTs) were performed in these. AR was evaluated at horse stable level based on faecal egg count reduction (FECR) and the lower limit of the 95% credible interval (LLCI) using the Bayesian hierarchical model. This study confirmed that strongylids (*Cyathostominae* (CYA)) resistant to FBZ are pervasive in Lithuania. FBZ was ineffective in three of the six stables (FECR 77.1–79.0%; 49.8–99.8 LLCI), was suspected to be ineffective in one stable (FECR 93.6%; 85.4–100 LLCI) and was effective (FECR 99.8–100%; 99.8–100 LLCI) in two stables. FEC showed a significant (P < 0.01) difference between the treatment and control groups. Only CYA larvae were detected in larval cultures derived from strongyle-positive faecal samples collected 14 days after treatment of a test group with FBZ.

**Conclusion:**

This in vivo study showed that resistance to FBZ in the treatment of strongyle nematodes is prevalent in horse stables in Lithuania. These findings should guide the implementation of more sustainable management of strongyle infections in horses in Lithuania.

## Background

Strongylid nematodes, particularly *Cyathostominae* (CYA), are ubiquitous in horse operations and are currently considered to be the main horse parasites at risk of developing anthelmintic resistance (AR), with the associated consequences of this on horse health [1, 2]. The spread of AR is in the focus for both parasitologists and horse practitioners around the world. As strongylid resistance has been recorded for all horse anthelmintics currently used [3], control of these infections has become challenging.

Anthelmintic resistance is characterised as a genetically transmitted loss of sensitivity to a formerly effective drug among the parasite population at the dose recommended by the manufacturer. The development of AR is based on the selection of specific alleles under drug pressure [4]. FBZ resistance is currently the rule rather than the exception in Europe [5–12] and other continents [13, 14]. Resistance to pyrantel (PYR) has progressively spread [4, 8, 11, 15, 16], but macrocyclic lactones (MLs) usually maintain sufficient efficacy. Early signs of resistance to MLs, such as shortened periods of egg reappearance [17, 18] or fully developed AR confirmed by faecal egg count reduction tests (FECRTs) have however been reported [8, 11].

Four anthelmintics belonging to three classes based on their chemical structure and pharmacological behaviour are used for controlling strongylid infections in Lithuania: Fenbendazol (FBZ), a benzimidazole (BZ); PYR, a tetrahydropyrimidine, and two MLs: ivermectin (IVM) and moxidectin (MOX). A nationwide study was performed in Lithuania in 2021 that evaluated IVM and PYR anthelmintics registered for the control of strongylid infections, and reported resistance to PYR and sufficient IVM efficacy [19]. However there are only limited data on the resistance of strongylids to FBZ drugs. The last confirmed resistance to BZ in Lithuania was in 2004, but that was in a narrow, small-scale study [20].

Therefore, the aim of this study was to provide further information about the efficacy of FBZ used against a population of strongylid parasites in horse stables in Lithuania.

## Methods

### Horses/animal selection

The study was conducted in March and November 2020 in six stables in Lithuania. Horses in these stables were used for sport, leisure riding and breeding. The initial screening included 121 horses, but only 89 horses met the inclusion criteria for further study that included strongyle faecal egg count (FEC) exceeding 200 eggs per gram (EPG). All the horses had access to pasture and had not received any antiparasitic treatment within the 8 weeks prior to the study.

### Evaluation of egg shedding

Pre-treatment and post-treatment faecal samples were taken from individual horses in a clean box, with samples manually collected from the top of the pile after spontaneous defecation. All the samples were immediately sealed by placing the faecal material in plastic rectal sleeves and tying a knot halfway up the sleeve. These were stored in a refrigerator (4 °C) and processed within 24 h.

### Egg-counting methods

The efficacy of FBZ was estimated using FECRT, the estimation of anthelmintic efficacy via post-treatment egg reduction [21, 22]. The Mini-FLOTAC technique [23] with the Fill-FLOTAC [24] was used following the protocol recommended for fresh herbivore faeces (5 g faeces and 45 mL flotation solution (NaCl) at a specific gravity of 1.28 and a multiplication factor of 5). This technique is based on the passive flotation of eggs in flotation chambers with total volumes of 1 mL and is characterised by a revolving reading disc that provides improved readability. The discs were examined by an experienced technician using microscope at a magnification of 100×. The eggs were then morphologically identified according to [25].

### Treatment

In each stable, the horses selected for testing were randomly assigned to two experimental groups. Group A was treated with FBZ (n = 10) and group B was the untreated control group (n ≥ 4). The number of animals in the respective treatment groups was based on the recommendation that 10 animals per group are considered sufficient for detecting differences in FEC between groups [26]. The weight of each animal was estimated using a girth measuring tape. The anthelmintic dosages and routes of application were in accordance with the drug manufacturer’s recommendations. FBZ (7.5 mg per kg body weight [BW]) was administered peros using the product Panacur (Intervet International B.V., The Netherlands). The anthelmintic was mixed with feed and administered orally.

### Differentiation of third‑stage larvae

Faecal samples collected in each stable on day 14 were pooled and processed for coproculture. A minimum of 3 g from each strongyle-positive sample was mixed and incubated for 7 days at room temperature in the laboratory (24–29 °C) (adding water to maintain an adequate moisture level and 4 g of vermiculite). Third-stage larvae (L3) were subsequently recovered from the coprocultures using the Baermann technique [27]. The L3 larvae were microscopically examined, differentiated by morphology characteristics, and identified according to ministry of agriculture, fisheries and food (MAFF) [28]. The first 100 L3 larvae, or all L3 if ≤ 100 developed L3 larvae, were identified per sample by the number, shape and arrangement of intestinal cells [29].

### Statistical analysis

Faecal egg count reduction (FECR) for individual horses was calculated by Bayesian hierarchical model analysis of the data using an estimate of mean FECR and 95% credible intervals (CIs) [30, 31]. FECR (%) was calculated for each horse, and mean FECRs, 95% CIs, and the means and ranges of FEC pre-treatment and FEC post-treatment were calculated for each operation. Data representing the anthelmintic efficacy in horses and particular operations are displayed in Table 1.

**Table 1:** Data for estimates of fenbedazole (FBZ) efficacy at stable level calculated using faecal egg counts (FEC)

Farm no.	Treatment group	n	FECpre (EPG)		FECpost (EPG)		FECR (95% CI)	
			Mean	Range	Mean	Range		
1	FBZ	10	989	320–2035	6	0–20	99.8% (98.8–99.9)	Efficient
	Control	4	533	240–1220	602	200–1475	NA	
2	FBZ	10	1002	540–1890	220	0-685	75.4% (53.8–98.8)	Resistance
	Control	7	625	210–1255	662	205–1880	NA	
3	FBZ	10	909	240–1720	60	0-200	93.6% (85.4–100)	Suspected resistance
	Control	4	842	260–1880	848	280–2020	NA	
4	FBZ	10	348	200–820	0	0	100% (100–100)	Efficient
	Control	5	265	220–890	331	265–1005	NA	
5	FBZ	10	782	220–1530	638	0-1465	79.0% (60.2–99.8)	Resistance
	Control	5	624	210–1265	731	260–1280	NA	
6	FBZ	10	789	220–1710	218	0-680	71.1% (49.8–98.6)	Resistance
	Control	4	689	340–890	720	420–920	NA	

*FBZ* fenbendazole, *FECpre* initial pre-treatment faecal egg count, *EPG* eggs per gram, *FECpost* post-treatment faecal egg count, *FECR* faecal egg count reduction

Drug efficacy (normal, suspected and reduced) at operation level was determined using mean FECR (%) and the lower limit of the 95% CIs (LLCI) [22, 32].

## Results

Faecal egg count pre-treatment was performed in six horse stables and on 121 horses, 19% of which can be considered low (0–195 EPG), 26% moderate (200–500 EPG) and 55% high (> 505 EPG) contaminators. Twenty-three horses were excluded from resistance testing because they had not met the inclusion criteria, therefore FECRTs were performed on the remaining 98 horses.

### Level of efficacy

A total of 60 horses were treated with FBZ and 29 horses were left untreated as the control group. FBZ demonstrated reduced efficacy (mean FECR 71.1–79.0%; 49.8–99.8 LLCI) in three horse stables, suspected reduced efficacy in one horse stable (mean FECR 93.6%; 85.4–100 LLCI) and demonstrated normal efficacies in two stables (FECR 99.8–100%; 98.8–100 LLCI) (Table 1). The results obtained in FBZ-treated horses were significant (P < 0.01). All the third-stage larvae isolated from strongyle egg positive faecal samples were identified as cyathostomin larvae.

### General efficacy

**Fig. 1:**
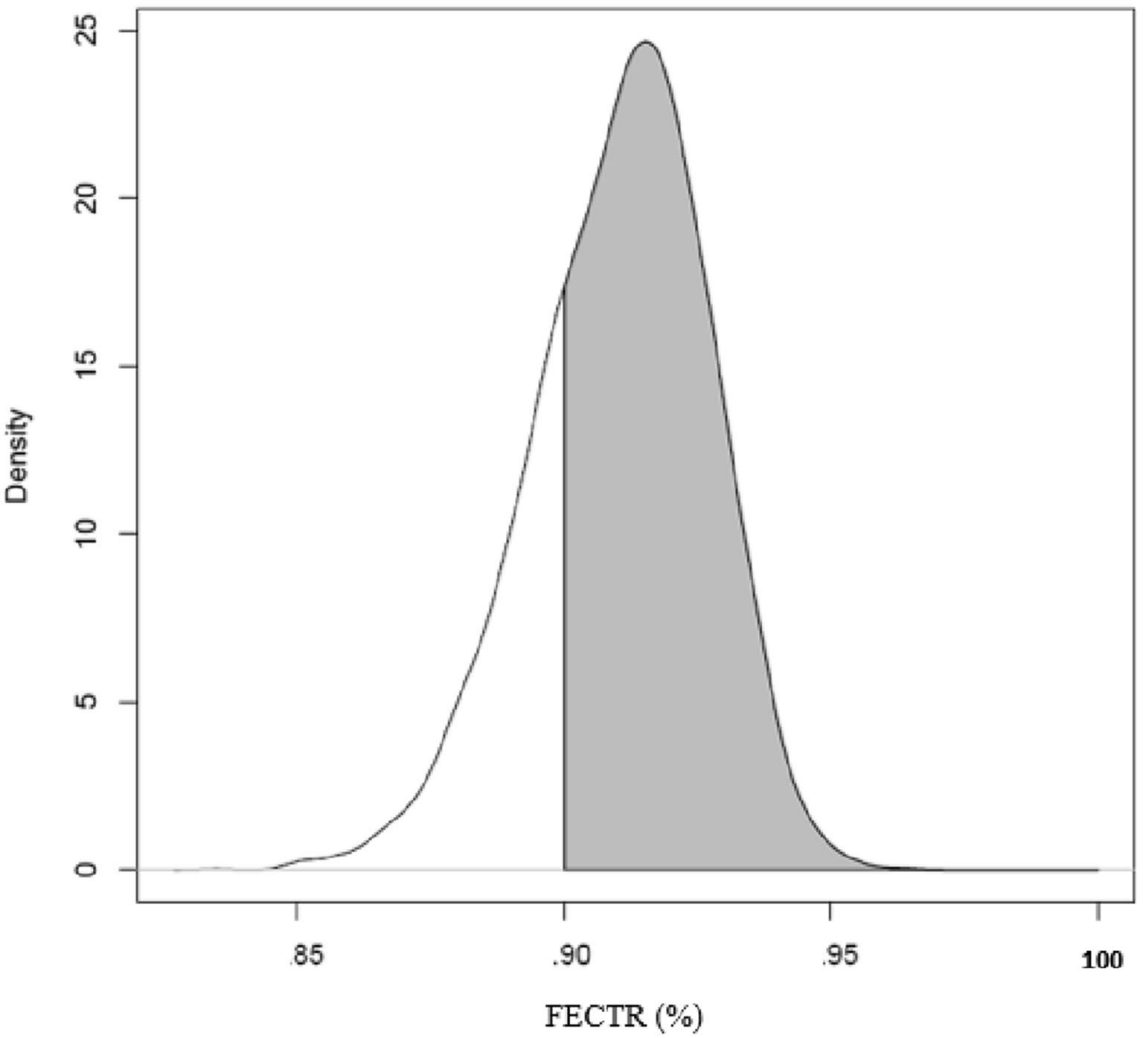
Posterior probability distribution of faecal egg count reduction (FECR%) for fenbendazole (FBZ) at horse level. FECTR%, faecal egg count reduction

Figure 1 presents the general efficacies of the anthelmintics visualised as the posterior distribution of faecal egg reduction. In the FBZ treatments, 0.92% (CI 0.88–0.94) resulted in sub-zero efficacies (individual FEC post-treatment exceeding FEC pre-treatment).

## Discussion

The horse industry in the Lithuania is growing, with the number of registered horses in 2021 exceeding 15,800. This study provides data on the efficacy of the FBZ anthelmintic compound used in Lithuanian horse stables.

All anthelmintics in Lithuania intended for use in horses are available by prescription only, and their distribution strictly relies on veterinary practitioners. Horses individually maintain their egg shedding potential, and the majority of eggs are produced by a small portion of herd individuals [33, 34]. In Lithuania, however, there continues to be a lack of a strategic approach comprising appropriate measures to determine the need to administer anthelmintics and verify their efficacy in order to avoid the drugs becoming ineffective. In this study, 19% of horses were considered to have a low parasitic burden as they shed fewer than 200 EPG. Most of the horses, however, still received the treatment along with all the other horses in their stables at fixed times throughout the year. In contrast, treatment twice a year could be insufficient for high shedders to avoid excessive contamination of pastures. The threshold for the selective-treatment approach has not been precisely determined for horses and could vary depending on individual conditions. Horses classified as moderate (200–500 EPG) and high (> 500 EPG) contaminators generally shed the majority of eggs and require anthelmintic treatment [21, 22].

This study confirmed that FBZ-resistant strongylids are pervasive in Lithuania. Only CYA were detected in larval cultures derived from strongyle-positive faecal samples collected 14 days after treatment with FBZ. Recent surveys show a decreasing prevalence of large strongyles in horse stables worldwide [7, 35–38], including Lithuania [19], while CYA are now considered the most important group of horse parasites [39, 40]. The prevalence of resistance to FBZ in Lithuanian horses detected in this study is similar to that recently described in other European countries such as Norway [41], Denmark [42] and the UK [43]. Small strongyles have been found to be resistant to FBZ in Finland (70%), indicating widespread resistance [16]. Geographically close to Lithuania, Varady et al. [6] have reported resistance to FBZ in Slovakia, with FECR values indicating resistance ranging from 65.1 to 86.3% in 14 horse stables. The last time resistance to BZ was confirmed in Lithuania was by Vyšniauskas et al. [20] in a modest sample study. Use of BZ in horses in Lithuania has been declining in recent years. In 2021 these anthelmintics accounted for less than 12% of the market share [44], which can be considered one of the factors influencing the results.

A high level of resistance to FBZ was confirmed in this study. The value of FECRT continues to decrease over time, and the incidence of individual sub-zero efficacies is increasing compared with previous studies [20]. Avoiding the use of FBZ to control strongylid infections is essential for preventing economic and health consequences. FBZ was launched on the Lithuanian market in 1976, and IVM was introduced 10 years later. Both anthelmintics were used under similar conditions, but FBZ lacks efficacy while IVM remains fully effective. Other factors possibly affecting AR need to be considered. Product formulation and packaging size may indirectly influence the exact dosage. Powder and granules (FBZ) mixed with grain are not willingly accepted by all horses and repeated underdosing may occur [45]. The dose of a drug in an applicator insufficient for standard warm-blooded animals (e.g. 450 kg BW mebendazole, a BZ) could tempt horse owners, for cost reasons, to administer only one paste to a horse requiring a larger amount. Finally, the variety of concurrently marketed products of the same anthelmintic class (e.g. FBZ and mebendazole) could substantially increase the use of one anthelmintic class with a false impression of rotation of anthelmintics with different modes of action [46].

Various approaches for analysing anthelmintic efficacy make the comparison of study results challenging. FECRT is currently a gold standard in AR detection, but it still has limitations such as low sensitivity, variable reliability of the coprological FEC methods used [47], the lack of standardisation and cut-off values for horses, and the difficulty of interpretation. The large variety of species of horse strongylids is also an important factor.

## Conclusion

This study provides comprehensive information about the current situation of the resistance of horse strongylids to FBZ anthelmintics in Lithuania. FBZ is no longer effective for the control of strongylids. Modern approaches to parasitic control, such as non-chemical or selective anthelmintic treatments, need to be implemented, but will require extensive education programmes for both horse owners and veterinarians. It is essential to identify and conduct further research on the risk factors that accelerate the development of AR.

## Data Availability

The datasets used and/or analysed during the current study are available from the corresponding author on reasonable request.
